# Acute lymphocytic myocarditis presenting as complete heart block in an adult: a case report

**DOI:** 10.1186/s43044-023-00406-w

**Published:** 2023-08-30

**Authors:** Thomas Camilleri, Neil Grech, Maryanne Caruana, Mark Sammut

**Affiliations:** 1 FY Training Program, Mater Dei Hospital, WF2G+PH6, Triq Dun Karm, Msida, MSD2090 Malta; 2 Department of Cardiology, Mater Dei Hospital, Msida, Malta

**Keywords:** Case report, Lymphocytic myocarditis, Acute myocarditis, Complete heart block, Pacemaker, Heart failure

## Abstract

**Background:**

Complete heart block (CHB) as a first presentation of acute viral myocarditis is a rare occurrence associated with increased morbidity and mortality. In such cases, an endomyocardial biopsy is recommended to make a clear histological diagnosis aiding to differentiate from other possible conditions such as sarcoiditic myocarditis, giant cell myocarditis, and eosinophilic myocarditis. Insertion of a permanent pacemaker may be considered on a case-to-case basis.

**Case presentation:**

A previously healthy 21-year-old female presented to the emergency department after having suffered two episodes of syncope on a background of a few days’ history of myalgias, chills, and rigors. Electrocardiogram showed high-grade Mobitz II block with intermittent periods of CHB. A bedside echocardiogram upon admission demonstrated normal biventricular systolic function. Given the patient’s unstable haemodynamic status and lack of obvious reversible causes for the CHB, a permanent dual-chamber pacemaker was inserted urgently. Initial blood investigations indicated an ongoing inflammatory process highlighting the possibility of myocarditis as a cause of the CHB. Therefore, a troponin level was taken and was noted to be elevated confirming the suspicion of myocarditis. The left ventricular ejection fraction (LVEF) decreased over the following days to approximately 20%, clinically resulting in pulmonary oedema and acute shortness of breath. The patient required aggressive intravenous diuresis and anti-heart failure medication. An endomyocardial biopsy (EMB) confirmed the diagnosis of lymphocytic myocarditis. The patient’s condition improved secondary to an improvement in LVEF and resolution of the heart block. A cardiac magnetic resonance (CMR) imaging performed 6 weeks from admission reported an improved LVEF of 51% with no late gadolinium enhancement (LGE). Based on the reassuring CMR findings and the resolution of CHB on follow-up pacemaker checks, it was deemed safe to explant the pacemaker.

**Conclusions:**

Acute myocarditis may be complicated with high-degree AV block and cardiogenic shock necessitating close observation in a critical care unit. A permanent pacemaker may provide atrio-ventricular synchrony which helps stabilise the patient’s condition and protect from a prolonged period of heart block. Early myocardial fibrosis on EMB and degree of LGE on CMR are indicators of persistent atrioventricular block. Guideline-directed treatment of heart failure is essential.

**Supplementary Information:**

The online version contains supplementary material available at 10.1186/s43044-023-00406-w.

## Background

Complete heart block (CHB) as a first presentation of acute myocarditis is a rare occurrence and is associated with an increased morbidity and mortality [1, 2]. We report a case of acute lymphocytic viral myocarditis presenting with CHB and subsequently complicated by acute heart failure.

## Case presentation

A previously healthy 21-year-old female presented to the emergency department after experiencing two episodes of syncope (the first while climbing stairs and the second while at rest). Prior to her presentation, she had been feeling unwell for several days with myalgias, chills, and rigors, but had no documented fevers. She gave no history of chest pain, breathlessness, or palpitations. Her only regular medication was a combined oral contraceptive pill, and she denied any recreational drug use.

At the time of the first clinical assessment, she was apyrexial, clammy, and lethargic and kept experiencing recurrent pre-syncopal episodes. The patient was euvolaemic with normal vesicular breath sounds, no lower limb oedema, and normal heart sounds. She was bradycardic with a heart rate of 40 bpm and hypotensive with a non-invasive blood pressure measurement of 95/45 mmHg.

## Investigations and treatment

**Fig. 1:**
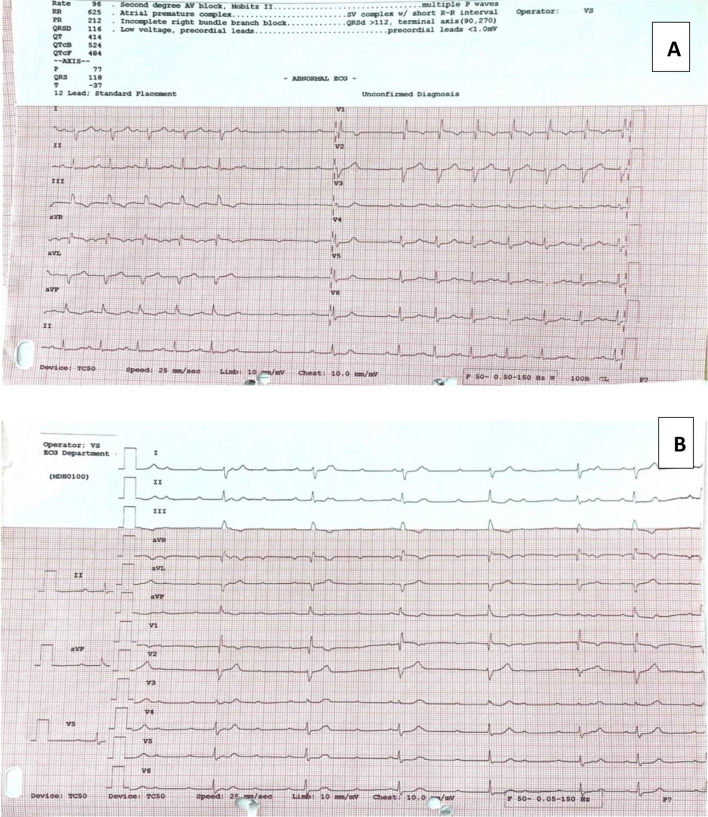
ECGs on admission. **A** ECG on admission showing high-grade Mobitz II block with intermittent complete heart block. **B** ECG on admission showing high-grade Mobitz II block with intermittent complete heart block with a narrow escape rhythm

Her electrocardiogram (ECG) showed Mobitz II second-degree AV block and intermittent episodes of CHB with a marginally wide ventricular escape rhythm (QRS 120 ms) at 40 bpm (Fig. 1). A bedside transthoracic echocardiogram (TTE) revealed normal biventricular size and function and normal valvular function. There was no pericardial effusion. Preliminary electrolyte levels on a venous blood gas sample and a chest X-ray were normal. Given the patient’s unstable haemodynamic status and lack of obvious reversible causes for her AV block, the patient was urgently taken for implantation of a permanent dual-chamber pacemaker from the emergency department.

Full blood results that became available later showed an ongoing inflammatory process with a C-reactive protein (CRP) level of 75 mg/l. Other blood counts revealed a microcytic anaemia with a haemoglobin of 10.7 g/dL and a normal white blood cell count (10 × 10^9^/L). A high-sensitivity troponin was taken due to the viral prodrome with an elevated CRP, indicating the possibility of myocarditis as a cause of the CHB. It was noted to be elevated at 976 ng/L (normal upper limit of 14 ng/L). An autoimmune and a serological viral screen were negative. Computed tomography (CT) coronary angiography excluded coronary artery disease as a cause for the myocardial injury.

On the 4th day of the patient’s admission, the atrioventricular block (AVB) resolved; however, her condition worsened due to a deterioration in LV systolic function with an ejection fraction of approximately 20–25% (Additional file 1: Video 1). A small circumferential pericardial effusion was also noticed on serial TTEs, although this was never of haemodynamic significance. She was started on low-dose colchicine (due to the pericardial effusion indicating pericardial inflammation), enalapril, spironolactone, and furosemide (targeting the patient’s LV systolic dysfunction). The patient’s troponin and N-terminal prohormone of brain natriuretic peptide (NTproBNP) levels peaked at 2057 ng/L on day 2 and 8674 pg/ml on day 6, respectively. Despite treatment, the patient developed cardiogenic shock with worsening dyspnoea, as well as pyrexia secondary to a lower respiratory tract infection. Anti-heart failure medication was omitted due to the hypotension, and broad-spectrum antibiotics were initiated. The patient was reviewed by intensive therapy unit (ITU) physicians; however, ITU admission was avoided after successfully offloading the pulmonary oedema with a furosemide infusion while maintaining oxygen saturations via normal face mask (delivering 10 L/min of oxygen). Inotropic support was not required and the patient’s condition stabilised.

**Fig. 2:**
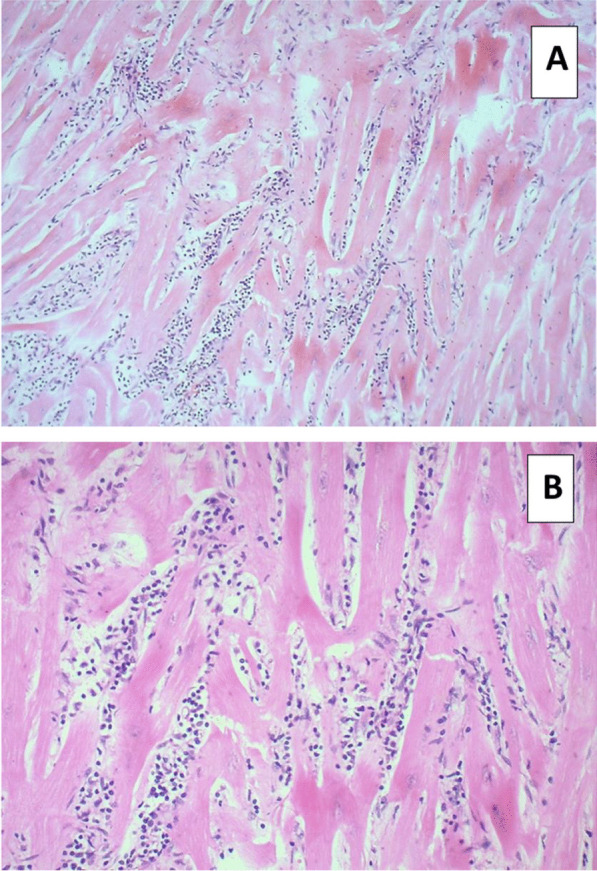
Endomyocardial biopsy findings. **A** Endomyocardial biopsy findings showing scanty interstitial lymphocytic inflammatory infiltrate. Stain used: H&E. Magnification: ×100. **B** Endomyocardial biopsy findings showing an extensive severe chronic inflammatory infiltrate consisting exclusively of lymphocytes surrounding myocytes with extensive myocyte destruction. Stain used: H&E. Magnification: ×200

In light of this deterioration, an endomyocardial biopsy (EMB) was performed. Three biopsies were taken from the right ventricular septum under fluoroscopic guidance with no complications. The biopsies revealed extensive severe inflammatory infiltrate consisting exclusively of lymphocytes surrounding myocytes with myocyte destruction, but no identifiable inflammatory giant cells, consistent with viral myocarditis (Fig. 2). Viral polymerase chain reaction (PCR) testing was performed for CMV, EBV, and enterovirus, all of which were negative.

## Differential diagnoses

The clinical presentation was indicative of myopericarditis (viral prodrome, elevated troponin, LV impairment, and pericardial effusion); however, it is uncommon for CHB to be associated with myocarditis. Given the rarity, other conditions were considered, including giant cell myocarditis, eosinophilic myocarditis, cardiac sarcoidosis, myocarditis exacerbating a pre-existing congenital AVB, and acute myocardial infarction.

Giant cell myocarditis is characterised by cytotoxic T cell-mediated destruction of cardiomyocytes, and it often follows a fulminant course which may be fatal unless the patient undergoes cardiac transplantation. Complete AVB is a well-known presenting feature of giant cell myocarditis and tends to affect young-to-middle-aged individuals, highlighting the importance of excluding this diagnosis on cardiac biopsy.

Eosinophilic myocarditis is also a potentially lethal condition caused by eosinophilic infiltration of the heart. However, this condition was unlikely given no prior history of other features of hypereosinophilia syndromes such as dermatological involvement. Cardiac sarcoidosis is a rare inflammatory granulomatous disorder which can infiltrate several organs including the heart and may subsequently cause arrhythmias and various degrees of AVB. A cardiac biopsy was performed in this case which excluded the above differential diagnoses.

The patient’s CHB recovered after a few days of admission making congenital AVB unlikely. Myocardial infarction was unlikely in this clinical case in view of the patient’s young age, absence of ischaemic risk factors, and atypical presentation. A CT coronary angiogram was performed which adequately excluded this diagnosis.

## Outcome and follow-up

The patient’s clinical condition stabilised gradually without the need for inotropic support and her LV ejection fraction increased to approximately 40% during admission (Additional file 2: Video 2). NTproBNP and troponin levels normalised within 2 months of admission (75 pg/ml and 14 ng/L, respectively). The heart block had since resolved and there was a return of her normal sinus rhythm. Subsequently, some changes were made to the pacemaker settings to avoid unnecessary pacing, namely a reduction in base rate to 50 bpm and sleep rate set at 45 bpm.

The patient was deemed well to be discharged home after a total of 13 days in hospital. The patient was discharged on anti-heart failure treatment including enalapril, carvedilol, and spironolactone. Low blood pressure limited the addition of empagliflozin and up titration of medication doses. Low-dose bumetanide was prescribed to maintain euvolaemia, and colchicine was also given for a total of 3 months as treatment of her associated pericarditis. Cardiac magnetic resonance imaging performed one month (allowing for the 6-week pacemaker insertion exemption period to elapse) following the patient’s discharge reported an LV ejection fraction of 51% with no signs of late gadolinium enhancement indicating the absence of ongoing myocardial inflammation (Additional file 3: Video 3). There was very minimal fluid in the pericardium. Follow-up pacemaker checks revealed no signs of high-degree AVB with good native rhythm. Based on the reassuring CMR result and pacemaker checks, it was deemed safe to explant her pacemaker in the following weeks.

## Discussion

Myocarditis is a broad term with various definitions and subtypes. In the 2013 position paper by the European Society of Cardiology (ESC), myocarditis is defined on the basis of histological, immunological, and immunohistochemical criteria [3]. This involves an array of investigations such as serological investigations (troponin, white cell count, C-reactive protein), an electrocardiogram, echocardiogram, and cardiac magnetic resonance imaging. However, the gold standard is EMB contributing towards a definite histological diagnosis [3–5]. Despite this recommendation, an EMB is usually limited to life-threatening situations or when the diagnosis is unclear. This is likely secondary to the risks involved, together with the decreasing availability of trained staff and equipment, as well as the increasing availability of CMR [6]. A statement paper by the American Heart Association recommends limiting the use of EMB to cases of an unexplained cardiomyopathy complicated by high degrees of block, ventricular arrhythmias, inotrope requirement, or failure to respond to medical treatment. If these are not present, then a CMR would suffice for the diagnosis [7]. In our case, an EMB was recommended based on both above guidelines and it was indeed essential as it provided a definite diagnosis while excluding other important differentials which may have required tailored therapy. A more recent expert consensus statement published in 2020 describes myocarditis with associated advanced AVB and LV impairment as complicated acute myocarditis, in keeping with our case [8]. In cases of fulminant myocarditis, LV impairment leads to cardiogenic shock requiring inotropic or mechanical circulatory support by definition, which was not required in our case [9].

The EMB reported a diagnosis of lymphocytic myocarditis which is characterised by CD3+ T lymphocyte and CD68+ macrophage infiltrates leading to myocyte inflammation and necrosis of varying degrees [5, 8]. The cause of the myocarditis in our case was assumed to be viral given the patient’s prodrome; however, there was no detected viral genome on polymerase chain reaction of the EMB. Having said this, a causative virus is only found in 30 to 40% of viral-induced lymphocytic myocarditis [10]. The results of autoimmune and toxic screens were normal, therefore excluding these factors in our case.

High-grade AVB is an uncommon finding in patients with acute lymphocytic myocarditis [2]. Varying degrees of AVB may occur in acute myocarditis due to ongoing inflammation resulting in myocardial interstitial oedema involving the conduction system [11]. As early as 1992, Morgera et al. [12] reported an incidence of 15.5% in active myocarditis. However, a more recent large observational study of 31,760 patients with myocarditis reported an incidence of high-grade AVB to be only 1.1% [1]. Given the rarity of high-grade AVB in myocarditis patients, other causes of AVB must be excluded. This may include cardiomyopathies (hypertrophic cardiomyopathy, other myocarditis types), electrolyte imbalances, hormonal deficiencies (such as hypothyroidism), and drug related [13]. All these differentials were sufficiently excluded on the basis of the adequate history taking, blood investigations, and the EMB. Indeed, AVB is more common in other types of myocarditis such as Lyme carditis, giant cell myocarditis, and sarcoiditic myocarditis. This complication has been reported in approximately 20–50% of these cases and indeed contributes significantly to an increased morbidity and mortality [10, 14, 15]. Apart from the higher incidence of AVB in these conditions, another early study noted that a pacemaker was required in 60% of cases with giant cell myocarditis, while only 8.3% of AVB in lymphocytic myocarditis required a pacemaker [16]. Permanent pacemaker insertion is rarely required in lymphocytic myocarditis with only a handful of cases reported [17–19]. A permanent pacemaker was inserted on presentation in our case due to several reasons. Firstly, it provided atrio-ventricular synchrony which helped stabilise the patient’s condition. This synchrony is not possible with a single lead temporary pacemaker. Furthermore, the outcome of the CHB is difficult to predict in cases of complicated acute myocarditis. It may take days to weeks to resolve and may possibly re-emerge once thought to be resolved. Indeed, monitoring the rhythm closely in the acute and subacute stages is essential. This may be done on ambulatory ECG monitoring or recordings from the pacemaker device as was done in our case. Early fibrosis of the myocardium in published cases seemingly played an important role in persistent high degree AVB and pacemaker dependence [18]. Therefore, CMR and EMB may aid in the decision for requirement of a permanent pacemaker based on fibrotic changes and a high degree of late gadolinium enhancement on CMR. In our case, the patient's AVB completely resolved in conjunction with CMR results (no late gadolinium enhancement or fibrosis), indicating a good prognosis.

Heart failure in acute myocarditis may lead to cardiogenic shock, hence the importance of guideline-directed heart failure treatment to assist in the myocardium recovery [20]. Inotropic and mechanical circulatory support may be required in cases of fulminant myocarditis [9]. In our case, inotropic support was not required and the patient’s LV recovered after a few weeks of anti-heart failure treatment.

## Conclusions

This case highlights the importance of prompt intervention in patients with acute myocarditis complicated with high degree AVB. It also highlights the possibility of rapid deterioration in LV systolic function necessitating close observation and monitoring in a critical care unit. It is reasonable to insert a permanent pacemaker as the duration and re-emergence of the block is difficult to predict while it also provides atrio-ventricular synchrony which helps stabilise patients' condition. Factors which may indicate a persistent block may include early myocardial fibrosis by EMB and degree of late gadolinium enhancement on CMR. Effective guideline-directed heart failure treatment is essential, aiming for a recovery in LV systolic function.

### Supplementary Information

**Additional file 1: Video 1.** Transthoracic echocardiogram with severely impaired LV systolic function. Transthoracic echocardiogram in parasternal long-axis, parasternal short-axis, and apical 4-chamber views demonstrating severely impaired left ventricular systolic function with particular hypokinesia of the septal wall. Small circumferential pericardial effusion may be seen. Pacemaker lead in-situ.

**Additional file 2: Video 2.** Transthoracic echocardiogram with improved LV systolic function. Transthoracic echocardiogram in parasternal long-axis, parasternal short-axis, and apical 4-chamber views demonstrating an improved left ventricular systolic function. A persistent small circumferential pericardial effusion is present. Pacemaker lead in-situ.

**Additional file 3: Video 3.** Cardiac magnetic resonance imaging in 2-chamber, 4-chamber, and short-axis views, demonstrating low-normal LV systolic function with an estimated ejection fraction of 51%. Artefact present due to the presence of the pacemaker and motion.

## Data Availability

Not applicable.

## Abbreviations

AVB: Atrioventricular block
CHB: Complete heart block
CMR: Cardiac magnetic resonance
CMV: Cytomegalovirus
CRP: C-reactive protein
CT: Computed topography
CXR: Chest X-ray
EBV: Epstein Barr Virus
ECG: Electrocardiogram
EF: Ejection fraction
EMB: Endomyocardial biopsy
ITU: Intensive therapy unit
LOC: Loss of consciousness
LV: Left ventricular
NTproBNP: N-terminal prohormone of brain natriuretic peptide
PCR: Polymerase chain reaction
TTE: Transthoracic echocardiogram
